# PARP16/ARTD15 Is a Novel Endoplasmic-Reticulum-Associated Mono-ADP-Ribosyltransferase That Interacts with, and Modifies Karyopherin-ß1

**DOI:** 10.1371/journal.pone.0037352

**Published:** 2012-06-11

**Authors:** Simone Di Paola, Massimo Micaroni, Giuseppe Di Tullio, Roberto Buccione, Maria Di Girolamo

**Affiliations:** Consorzio Mario Negri Sud, Santa Maria Imbaro (Chieti), Italy; Ecole Polytechnique Federale de Lausanne, Switzerland

## Abstract

**Background:**

Protein mono-ADP-ribosylation is a reversible post-translational modification that modulates the function of target proteins. The enzymes that catalyze this reaction in mammalian cells are either bacterial pathogenic toxins or endogenous cellular ADP-ribosyltransferases. The latter include members of three different families of proteins: the well characterized arginine-specific ecto-enzymes ARTCs, two sirtuins and, more recently, novel members of the poly(ADP-ribose) polymerase (PARP/ARTD) family that have been suggested to act as cellular mono-ADP-ribosyltransferases. Here, we report on the characterisation of human ARTD15, the only known ARTD family member with a putative C-terminal transmembrane domain.

**Methodology/Principal Findings:**

Immunofluorescence and electron microscopy were performed to characterise the sub-cellular localisation of ARTD15, which was found to be associated with membranes of the nuclear envelope and endoplasmic reticulum. The orientation of ARTD15 was determined using protease protection assay, and is shown to be a tail-anchored protein with a cytosolic catalytic domain. Importantly, by combining immunoprecipitation with mass spectrometry and using cell lysates from cells over-expressing FLAG-ARTD15, we have identified karyopherin-ß1, a component of the nuclear trafficking machinery, as a molecular partner of ARTD15. Finally, we demonstrate that ARTD15 is a mono-ADP-ribosyltransferase able to induce the ADP-ribosylation of karyopherin-ß1, thus defining the first substrate for this enzyme.

**Conclusions/Significance:**

Our data reveal that ARTD15 is a novel ADP-ribosyltransferase enzyme with a new intracellular location. Finally, the identification of karyopherin-ß1 as a target of ARTD15-mediated ADP-ribosylation, hints at a novel regulatory mechanism of karyopherin-ß1 functions.

## Introduction

Mono-ADP-ribosylation is a covalent, post-translational modification catalysed by bacterial toxins and eukaryotic ADP-ribosyltransferases. These enzymes transfer the ADP-ribose moiety from ß-NAD+ to specific amino acids of various cellular acceptor proteins, and as a consequence affect their biological function [1], [2], [3]. ADP-ribosylation was originally identified as the pathogenic mechanism of certain bacterial toxins: the diphtheria, cholera, pertussis and clostridia toxins are in fact mono-ADP-ribosyltransferases known to cause various pathologies as a consequence of their translocation into mammalian host cells [4], [5]. In mammals, enzymes structurally and functionally related to these toxins have been identified and characterized as intracellular or extracellular ADP-ribosyltransferases (ART) [2], [6]. These two groups of mammalian ARTs are defined as ARTC (Clostridia-toxin-like) and ARTD (Diphtheria-toxin-like), respectively [7]. The ARTC family includes glycosylphosphatidylinositol (GPI)-anchored and secreted enzymes that lead to extracellular mono-ADP-ribosylation [6], [8], [9]. Four different human ARTCs have been identified (ARTC1, 3, 4, 5) of which ARTC1 and ARTC5 are active enzymes that modify the arginine residues of secreted and plasma membrane-associated proteins, such as human neutrophil protein 1 (HNP1) and integrin-α7 [10], [11], [12]. Intracellular targets of mono-ADP-ribosylation have also been described [13], [14], [15], but only in one case (glutamate dehydrogenase; GDH) has the enzyme involved actually been identified: SirT4 [16]. This enzyme mono-ADP-ribosylates mitochondrial GDH thus repressing its activity [16] and consequently regulating insulin secretion in pancreatic ß cells. SirT4 is a member of a third NAD+-using family of proteins, the sirtuins, which encode protein deacetylases [17], [18]. The mammalian mono-ADP-ribosyltransferases responsible for intracellular mono-ADP-ribosylation are only now beginning to be identified. In addition to the sirtuins SirT4 and SirT6, novel members of the poly-ADP-ribose polymerase (PARP/ARTD) family are also being implicated in intracellular mono-ADP-ribosylation [2], [19], [20]. The human ARTD family includes six members (ARTD1-6), which are typical poly-ADP-ribosyl polymerases and eleven novel poorly characterized members (ARTD7-17) [19], [20]. The typical PARPs can transfer multiple ADP-ribose residues, and even branched polymers of ADP-ribose, onto their target proteins, thus regulating DNA repair, apoptosis and chromatin dynamics [19]. PARP1/ARTD1, the founding member of this family, acts as a molecular sensor of DNA breaks and plays a key role in the spatial and temporal organisation of their repair [21], [22]. It catalyzes both intermolecular auto-modification and hetero-modification of histones or proteins involved in DNA synthesis and repair [21], [22], [23]. PARP/ARTD 2-6, are also poly-ADP-ribosyl polymerases involved in DNA repair (ARTD2 and ARTD3), regulation of telomere length (ARTD5 and ARTD6), spindle pole function (ARTD3, ARTD5 and ARTD6) and genotoxic response (ARTD4) [24], [25], [26], [27], [28]. These polymerases are characterised by the H-Y-E triad of amino-acid residues in the catalytic domain, while the most recently identified members, ARTD7 to ARTD17, feature variations of this motif and are unlikely to promote the formation of ADP-ribose polymers, despite the overall similarity of the catalytic domain [19], [20]. Some of these enzymes have been proposed to act as cellular mono-ADP-ribosyltransferases, and indeed this has been demonstrated for ARTD10 [29]. Little is known concerning the biological roles of these enzymes, and only fragmented information has been obtained during recent years [30], [31], [32], [33], [34], [35].

**Figure 1:**
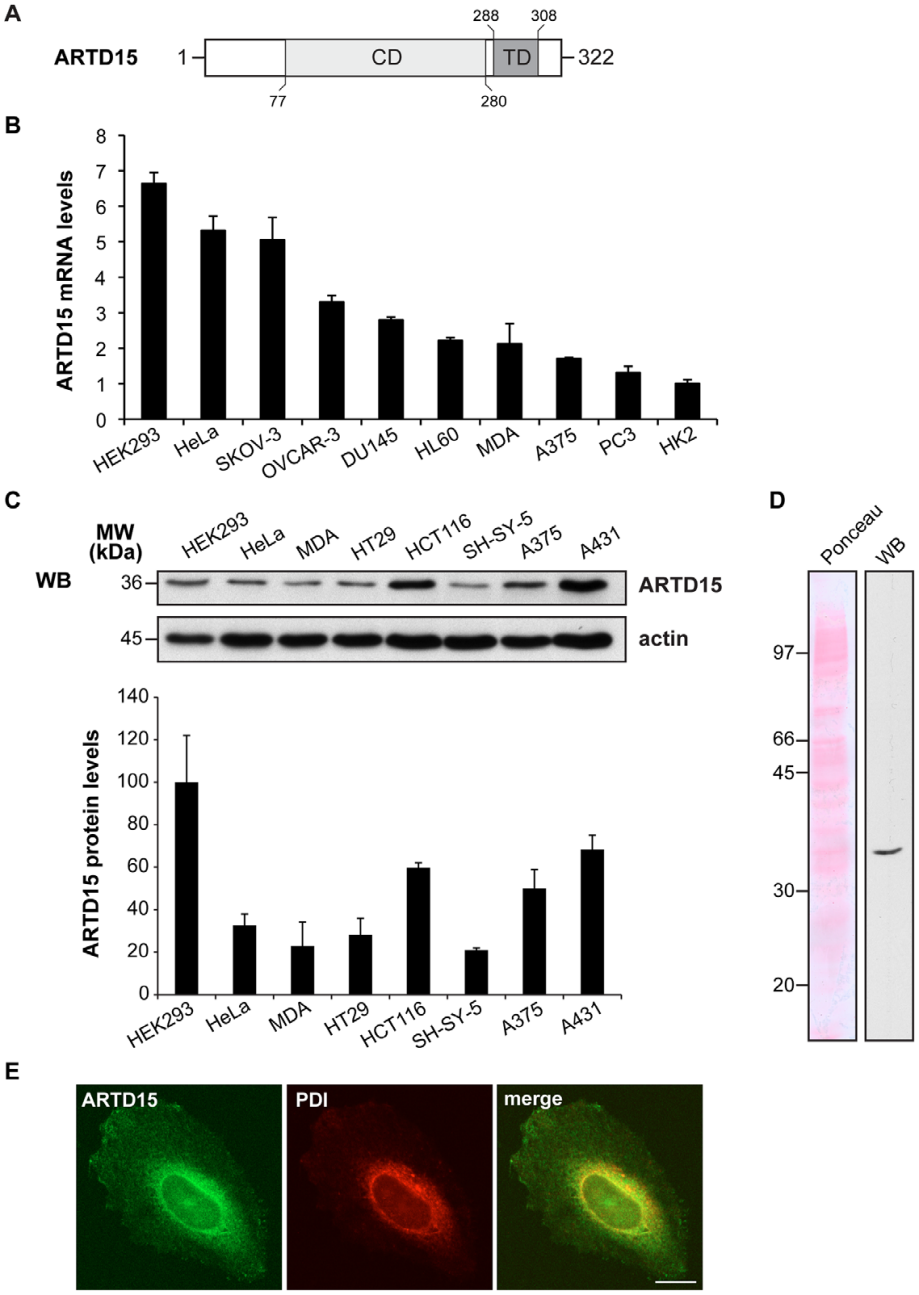
ARTD15 is expressed in different human cell lines. (**A**) Schematic diagram of ARTD15 domain structure. CD: catalytic domain; TD: transmembrane domain. (**B**) Levels of ARTD15 transcript determined by qRT-PCR, normalized to GAPDH RNA and then reported as arbitrary units relative to the ARTD15 transcript in HK2 cells (taken as 1). Data shown represent the mean (±SD) of two independent experiments performed in triplicate. (**C**) Expression of ARTD15. Western blotting (WB) showing endogenous ARTD15 protein and actin levels in 50 µg protein from total cell lysates. The expression levels of the ARTD15, normalized to actin, are shown in the histogram relative to those of HEK293 cells (taken as 100). Data shown represent the mean (±SD) of two independent experiments performed in triplicate. (**D**) Ponceau S staining and Western blotting (WB) of 100 µg protein from total HEK293 cell lysate are shown. (**E**) Immunofluorescence staining of endogenous ARTD15 (green) in combination with PDI (red) shows co-localization of ARTD15 protein with the ER. Bar, 20 µm.

**Figure 2:**
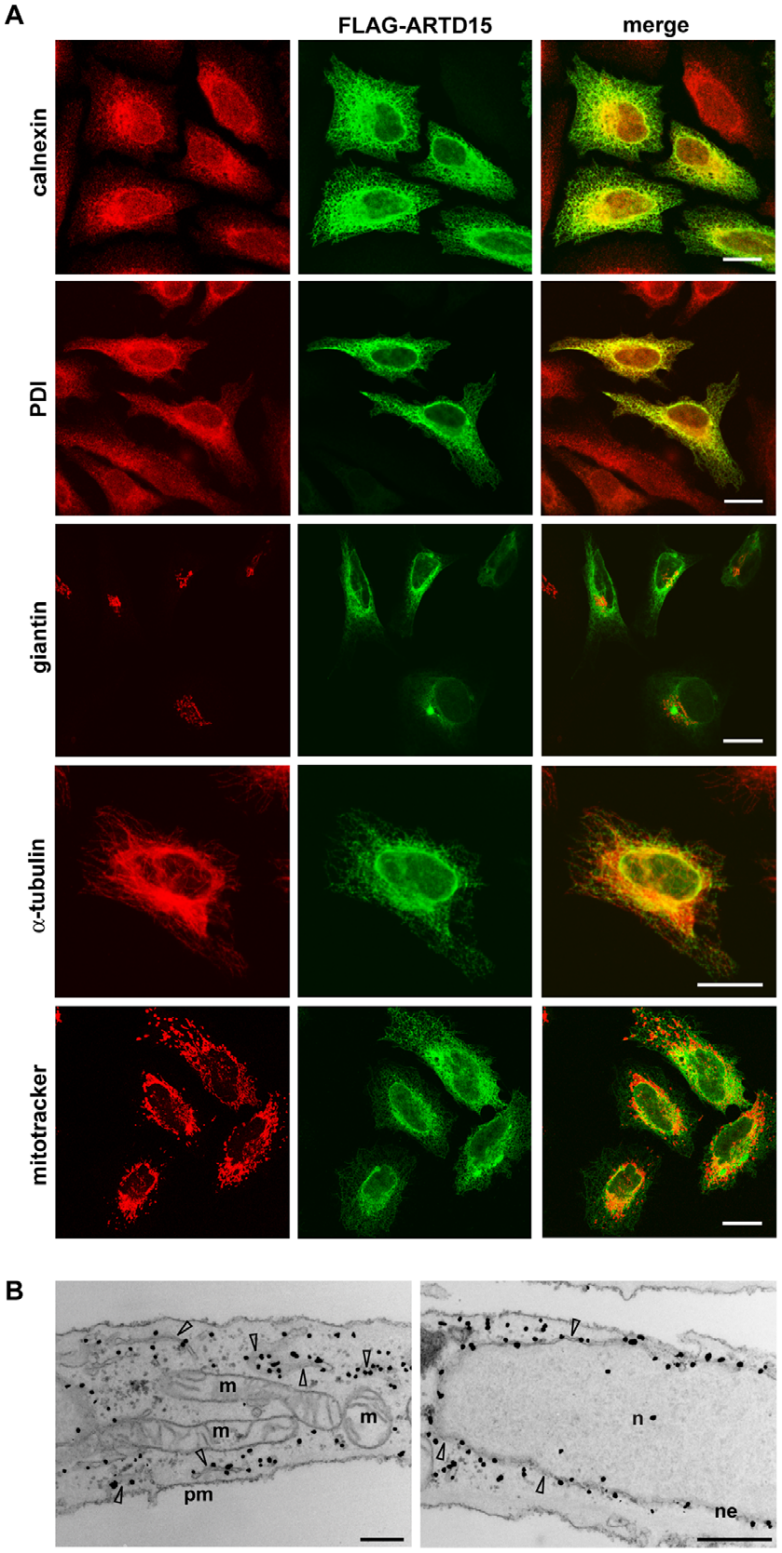
ARTD15 is an ER resident protein. HeLa cells were transfected with FLAG-ARTD15. (**A**) Immunofluorescence staining of ARTD15 (green) in combination with the indicated markers (red) shows co-localization of ARTD15 protein with the ER. Bar, 20 µm. (**B**) Electron microscopy analysis of immuno-gold staining of ARTD15 (black dots). Arrows show ER tubular structures and nuclear envelope; cellular organelles are indicated by abbreviations (n: nucleus; pm: plasma membrane; m: mitochondria; ne: nuclear envelope). Bar, 500 nM. Data shown are representative of three independent experiments.

Karyopherin-ß1/importin-ß1 (Kapß1, uniprot ID Q14974) plays a pivotal role in the shuttling of proteins with nuclear localisation signals (NLSs), between the cytosol and the nucleus, through the nuclear pore complex (NPC) [36], [37], [38]. During this process, Kapα (karyopherin-α/importin-α), binds to the NLS, whereas Kapß1 binds directly to Kapα, leading to the formation of a tri-molecular complex [39], [40], [41]. The complex tethers to, and passes through the NPC by Kapß1 binding to the nucleoporins. Once in the nucleoplasm, the complex releases the cargo protein, and Kapß1 and Kapα are exported back to the cytosol to re-initiate a new import cycle [37], [38]. In addition to its role in nuclear transport, Kapß1 has also been shown to participate in regulating mitotic spindle formation [42], [43]. Specifically, Kapß1 acts as a negative regulator of NuMA and TPX2, two microtubule-associated proteins involved in the organisation of the mitotic spindle [44], [45].

Here we report on the characterization of human PARP16/ARTD15 (herein referred to simply as ARTD15), the only known member of the family that does not contain recognizable accessory domains beyond the catalytic one. ARTD15 is a 36-kDa protein containing a predicted C-terminal transmembrane tract, but no information was available concerning its intracellular localisation and biochemical properties. We now show that ARTD15 is an endoplasmic reticulum-associated type IV enzyme with mono-ADP-ribosyltransferase activity, able to interact with, and mono-ADP-ribosylate the nuclear transport factor Kapß1.

**Figure 3:**
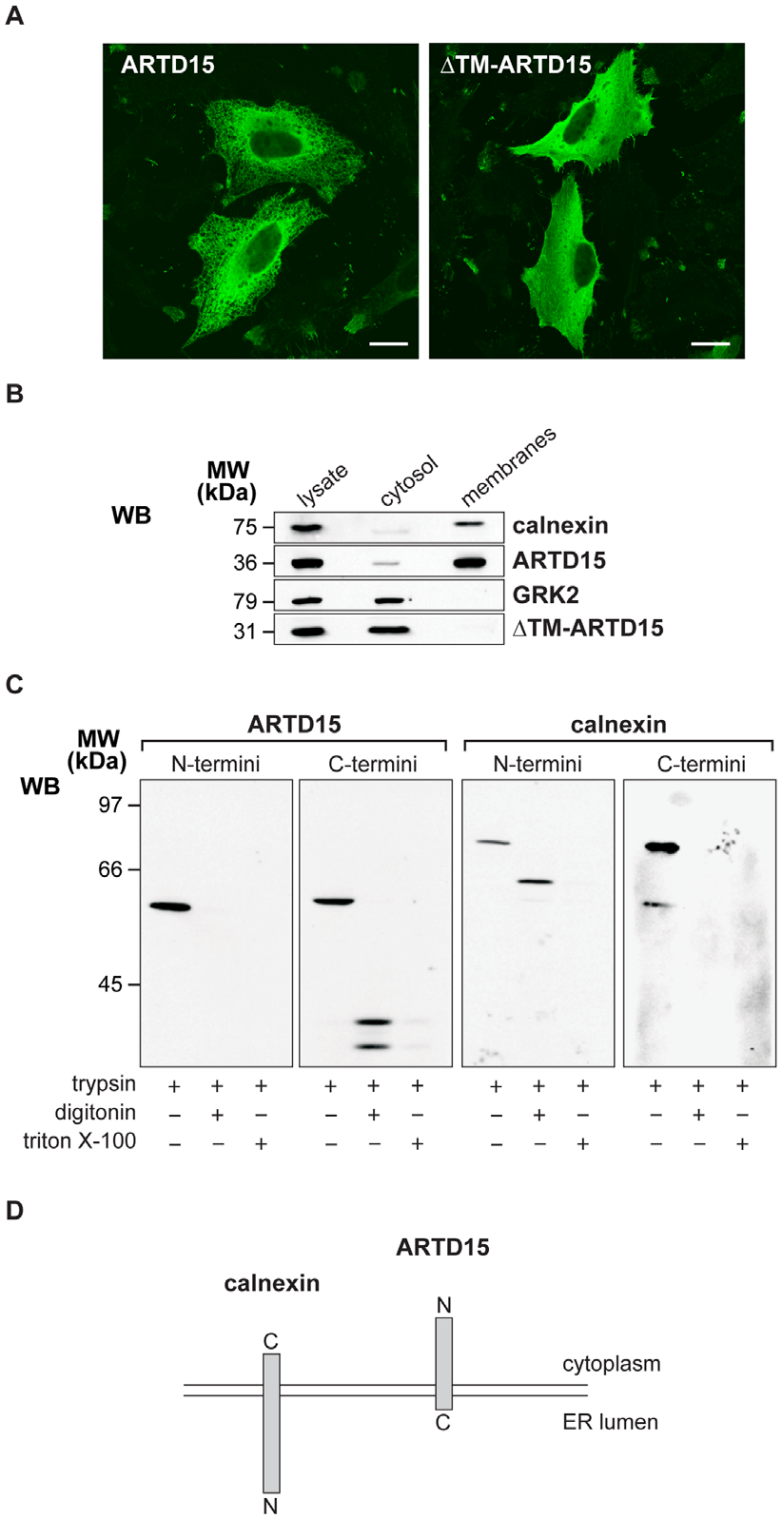
ARTD15 is an ER tail-anchored protein. HeLa cells were transiently transfected with full-length (ARTD15) or deleted (ΔTM-ARTD15) FLAG-ARTD15 and analyzed either by (**A**) immuno-fluorescence microscopy with an anti-FLAG antibody; Bar, 20 µm or by (**B**) Western blotting of total lysates (60 µg), cytosol and total membrane (30 µg) proteins using anti-FLAG antibody to visualize ARTD15 and anti-GRK2 and anti-calnexin antibodies as a control of cell fractionation. (**C**) Protease protection assay performed with HeLa cells transfected with N-termini or C-termini GFP-ARTD15. Western blotting of ARTD15 revealed with an anti-GFP antibody, and of calnexin revealed with antibodies raised against the N-termini or C-termini of calnexin. (**D**) Schematic representation of ARTD15 (based on our results) and calnexin protein orientation. The N-termini (N) and C-termini (C) of the proteins with respect to the endoplasmic reticulum are indicated. The data shown in A, B, and C are representative of at least three independent experiments.

## Results

### ARTD15 is Expressed in Numerous Human Cell Lines

ARTD15 with its predicted 323 amino acids is the smallest member of the PARP/ARTD family. Unlike other ARTDs, ARTD15 does not contain recognizable accessory domains and only features the catalytic domain (Fig. 1A).

We first determined if the ARTD15 gene was transcribed in various cell lines by quantitative real-time PCR (qRT-PCR). The highest mRNA levels were found in HEK293 and HeLa cell lines (Fig. 1B). Some of these cell lines were further analysed for protein expression using an antibody raised against ARTD15 (Fig. 1C, top; Fig. 1D). The protein expression levels of endogenous ARTD15 were measured relative to those in HEK293 (Fig. 1C, bottom panel). This is the first evidence that ARTD15 is transcribed and that the protein is ubiquitously expressed.

**Figure 4:**
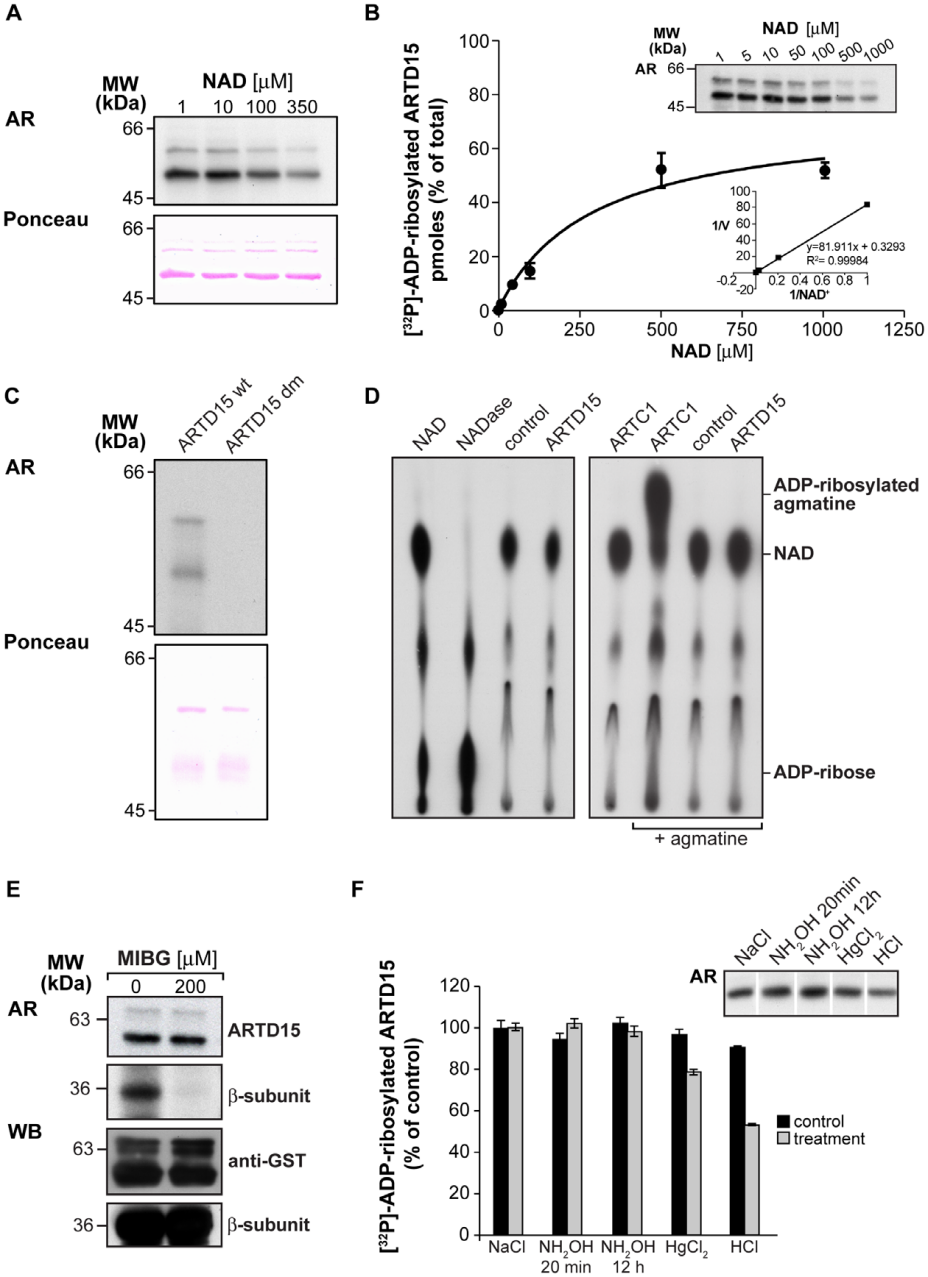
ARTD15 is an active ADP-ribosyl transferase. (**A & C**) Two µg of recombinant, purified GST-ARTD15 or GST-ARTD15-H152A/Y254A (ARTD15 dm) were [^32^P]-ADP-ribosylated *in vitro* in the presence of increasing concentration of NAD (as indicated in **A**) or with 10 µM NAD (**C**) and analysed by autoradiography (AR). Ponceau red staining is a control of protein loading. (**B)** Recombinant, purified GST-ARTD15 (300 ng) was [^32^P]-ADP-ribosylated and analysed by autoradiography (AR). The graph indicates the picomoles of [^32^P]-ADP-ribosylated ARTD15. The inset shows the Lineweaver-Burk analysis of the data. The data shown are the means (±SD) of four independent experiments performed in triplicate. (**D**) Total membrane proteins (50 µg) obtained from HeLa cells transiently transfected with empty vector (control), ARTD15 or ARTC1, as indicated, were [^32^P]-ADP-ribosylated *in vitro* in the absence (left) or presence (right) of 1 mM agmatine. The supernatants and, as a further control, [^32^P]-NAD (NAD) and [^32^P]-ADP-ribose (NADase) were analysed by TLC and autoradiography. The data shown are representative of at least five independent experiments. (**E**) Two µg of recombinant, purified GST-ARTD15 (ARTD15) or plasma membranes added with 250 ng of purified ßγ dimer (ß subunit) were ADP-ribosylated with [^32^P]-NAD^+^ for 1 h at 37°C, in the presence or absence of MIBG. The loading control is shown (WB). Data shown are representative of at least three independent experiments. (**F**) ADP-ribosylated ARTD15 was blotted and the filters were treated with the indicated compounds. Data reported in the graph are the mean (±SD) of three independent experiments performed in duplicate (control: untreated sample). The inset shows a representative experiment.

### ARTD15 is a Transmembrane Protein That Localises to the Endoplasmic Reticulum

ARTD15 is the only PARP/ARTD family member with a predicted C-terminal transmembrane (TM) domain (S288–I308; Fig. 1A). To investigate the cellular localisation of human ARTD15 we analyzed both endogenous (Fig. 1E) and over-expressed (Fig. 2A) protein by immunofluorescence. A clear peri-nuclear staining of endogenous ARTD15 and its co-localization with the endoplasmic reticulum (ER) protein disulfide isomerase (PDI) is shown in Figure 1E. Next, we generated an N-terminus FLAG-tagged ARTD15 (FLAG-ARTD15), and transiently expressed it in HeLa cells that were then analyzed by immunofluorescence, immuno-electron microscopy (EM) and Western blotting. Immunofluorescence images confirmed peri-nuclear ARTD15 localisation (Fig. 2A), while there was no detectable FLAG staining in mock-transfected cells. Moreover, over-expressed ARTD15 co-localised with the ER proteins calnexin and PDI (Fig. 2A), but not with markers of other intracellular compartments, such as the Golgi complex, mitochondria and microtubules (Fig. 2A). Immunogold labelling and electron microscopy confirmed ARTD15 localisation to ER and further highlighted the association with tubular ER structures and to the nuclear envelope (Fig. 2B). These results demonstrate that ARTD15 resides in the ER.

**Figure 5:**
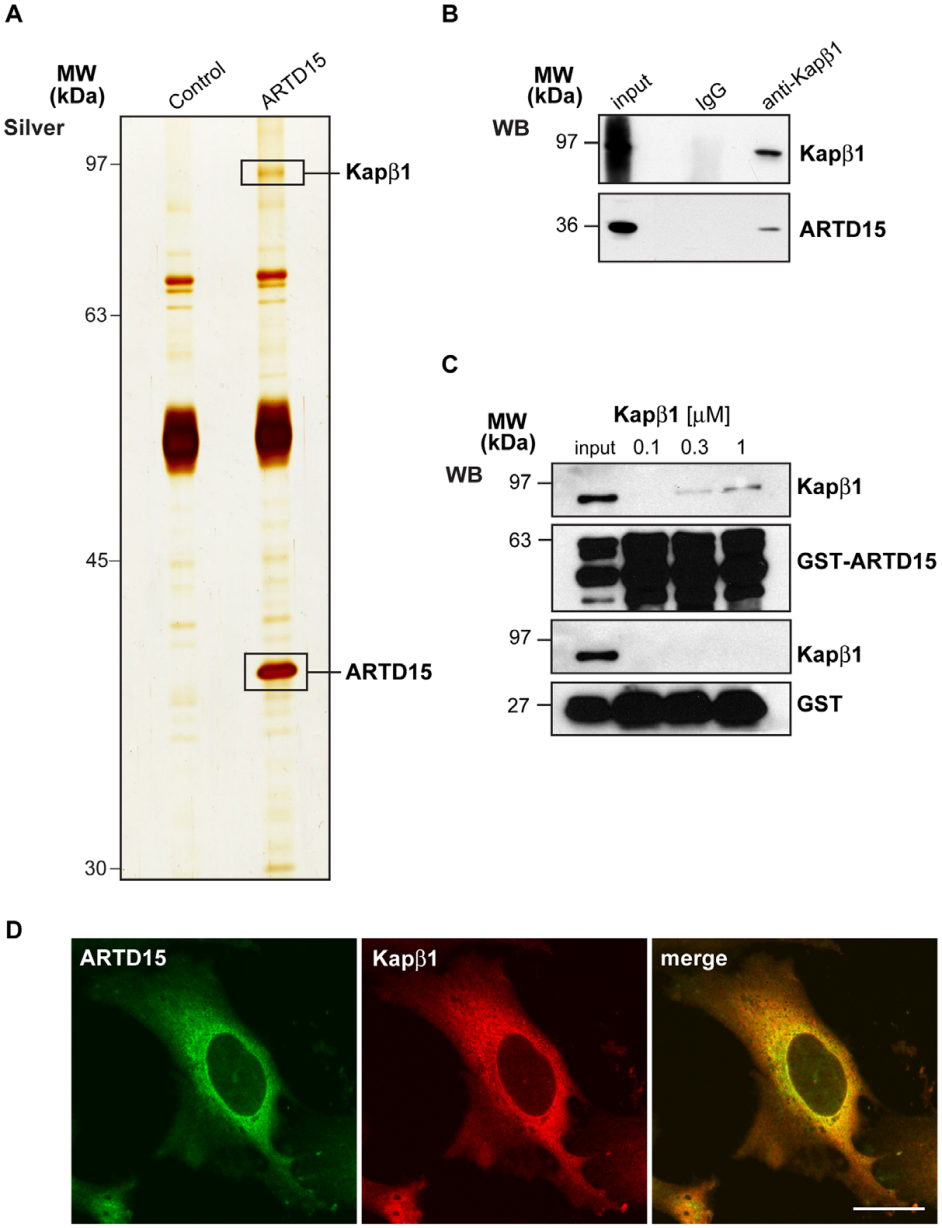
ARTD15 is a Kapß1 interactor. (**A**) Lysates (6 mg protein) from HeLa cells transfected with empty vector (control) or FLAG-ARTD15 were immunoprecipitated with a polyclonal anti-FLAG antibody. Proteins were separated by 10% long SDS-PAGE and proteins revealed by silver staining. Differential proteins were excised from the gel and identified by MALDI-ToF mass spectrometry (boxed bands). (**B**) Cell lysates from HeLa cells (10^6^ cells/assay) transfected with FLAG-ARTD15 were immunoprecipitated with an anti-Kapß1 antibody or with control IgG. The input is shown (1/20 of the total sample). (**C**) GST or GST-ARTD15 (0.1 µM) were incubated with increasing amounts of His-Kapß1. GST proteins were pulled down with gluthathione resin. Precipitated Kapß1 protein was probed with an anti-His antibody. (**D**) Immunofluorescence staining of endogenous ARTD15 (green) and endogenous Kapß1 (red) in HeLa cells. Bar, 20 µm.

**Table 1:** Protein identification.

NCBI acc. number	Name	Score	Matched peptide, no.	Sequence coverage, %	Molecular mass, kDa
AAH36703.1	Kapß1	127	13	20	97
AAH06389.1	ARTD15	96	9	32	36

Protein scores ≈ 65 are significant (*P*<0.05).

To further investigate the intracellular localization of ARTD15, we generated a deletion mutant lacking the region encompassing the putative transmembrane domain (aminoacids 278–322; FLAG-ΔTM-ARTD15). FLAG-ARTD15 and FLAG-ΔTM-ARTD15 were thus transiently expressed in HeLa cells and the sub-cellular localisation of the mutated proteins analyzed by immunofluorescence (Fig. 3A). FLAG-ΔTM-ARTD15 showed a diffuse, cytosolic localization compared with full length ARTD15 (Fig. 3A). The cytosolic localization was then confirmed by Western Blot analysis (Fig. 3B), which confirmed that FLAG-ΔTM-ARTD15 was confined to the cytosolic fraction as opposed to the full length protein, which was found in total membranes (Fig. 3B). Altogether, these results demonstrate that the C-terminal region, containing the predicted transmembrane domain, is necessary and sufficient for the localisation of ARTD15 to ER membranes.

**Figure 6:**
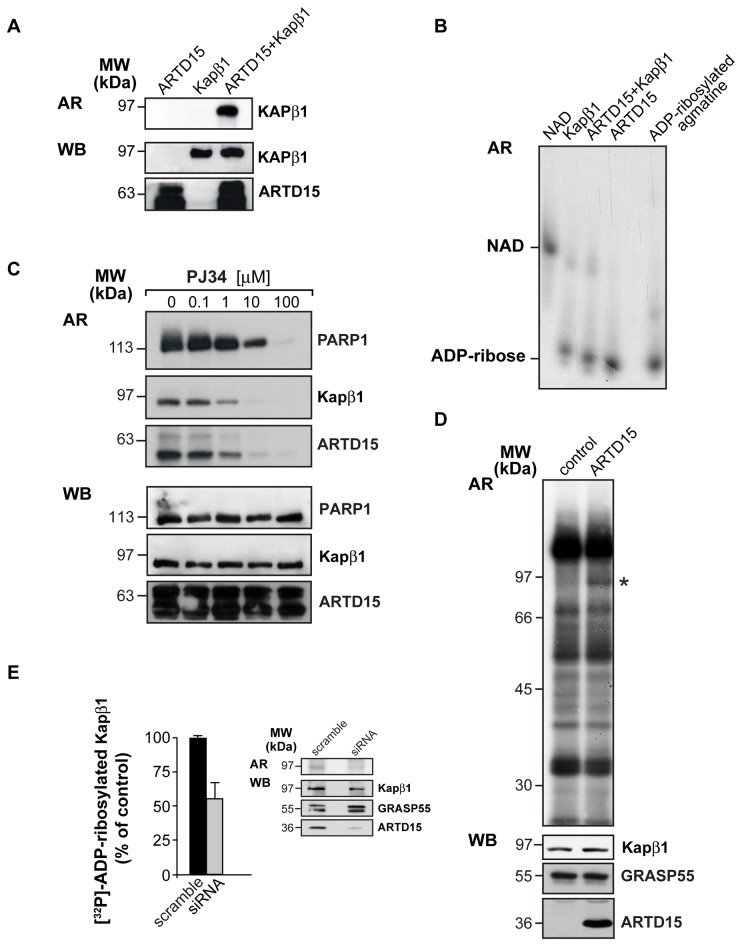
ARTD15 catalyses Kapß1 mono-ADP-ribosylation. (**A**) His-Kapß1 was [^32^P]-ADP-ribosylated *in vitro* for 6 hours at 37°C in presence of GST-ARTD15, separated by SDS-PAGE and analysed by autoradiography (AR). Recombinant proteins are shown by immunoblotting with anti-GST (ARTD15) and anti-His antibodies. (**B**) [^32^P]-ADP-ribosylation products derived from [^32^P]-ADP-ribosylated GST-ARTD15, His-Kapß1 and from [^32^P]-ADP-ribosylated agmatine and [^32^P]-NAD, as controls, were analysed by high-resolution PAGE to visualise ADP-ribose chain length. (**C**) GST-ARTD15 and His-Kapß1 were [^32^P]-ADP-ribosylated *in vitro* for 6 hours at 37°C in presence of 10 µM NAD, in presence of increasing concentration of the PARP1 inhibitor PJ34 (0.1–100 µM), separated by SDS-PAGE and analysed by autoradiography (AR). [^32^P]-ADP-ribosylated PARP1 was used as control. Immunoblotting with anti-PARP1, anti-GST (ARTD15) and anti-His (Kapß1) showed loaded protein. (**D**) Total membranes (50 µg) from HeLa cells transfected with empty vector (control) or ARTD15 were [^32^P]-ADP-ribosylated *in vitro* in presence of 10 µM NAD. Solubilised proteins were analysed by SDS-PAGE and autoradiography (AR). The single differential target (*) was recognized with an anti-Kapß1 specific antibody (WB). WB also shows over-expressed ARTD15, and the loading control GRASP55. The data shown are representative of five to ten experiments. (**E**) Quantification of [^32^P]-ADP-ribosylated Kapß1 in total membranes prepared from both control (scrambled) and ARTD15-silenced Hela cells. The data are means (±SD) of three experiments. The inset shows an example of the inhibition of Kapß1 ADP-ribosylation (AR) and of the ARTD15 knock-down (WB). WB also shows Kapß1, and GRASP55 as a loading control.

To analyse the orientation of ARTD15, we applied a trypsin protease-protection assay (see Materials and Methods). To this end we generated N-terminal and C-terminal GFP-tagged ARTD15 fusion protein. HeLa cells were transiently transfected with these constructs and were either left untreated (control), or incubated with digitonin to permeabilise the plasma membrane, or with Triton X-100 to solubilise all cellular membranes. Cells were then treated with trypsin, lysed, and finally analyzed by WB using an anti-GFP antibody to reveal ARTD15 and two antibodies directed against either the N- or C-terminus of calnexin (as a control). Calnexin is a well-characterised ER type I transmembrane domain protein with its N-terminal end facing the lumen and the C-terminal portion in the cytoplasm [46]. As expected, the C-terminal of calnexin was no longer detected when trypsinised after digitonin permeabilisation, whereas its N-terminus was lost only after Triton X-100 solubilisation, which allows trypsin to enter the ER lumen (Fig. 3C; calnexin). In the case of ARTD15 instead, trypsinisation resulted in the loss of its N-terminal end after mild permeabilisation with digitonin (Fig. 3C; ARTD15). Thus the N-terminus of ARTD15 is exposed to the cytoplasm, whereas its C-terminus faces the ER lumen. Altogether, these data demonstrate that, as opposed to calnexin, ARTD15 is a single pass transmembrane protein with the N-terminal region (aa. 1–280) positioned toward the cytoplasm, and the very short C-terminal tail (aa. 300–322) facing the ER lumen (Fig. 3D). These results define ARTD15 as a tail-anchored (type IV) protein.

**Table 2:** List of primers used in this study.

Primer name	Primer sequence
***ARTD15HindFor***	GCGAAGCTTATGCAGCCCTCAGGCTGG
***ARTD15BamRev***	GCGGGATCCTCTTTTCGCACGATTCCAAAAG
Δ***TM-ARTD15BamRev***	GCGGGATCCCTTGGGTGGCTTCTGTGAATACAC
***ARTD15EcoFor***	GCGGAATTCATGCAGCCCTCAGGCTGG
***ARTD15SalRev***	GCGGTCGACTCTTTTCGCACGATTCCAAAAG
***ARTD15H152AFor***	CGAGACCTAATCTATGCATTTGCTGGTAGCCGCCTAGAAAACTTC
***ARTD15H152ARev***	GAAGTTTTCTAGGCGGCTACCAGCAAATGCATAGATTAGGTCTCG
***ARTD15Y254AFor***	GAGACATCCCTCCCAAGGCCTTCGTGGTCACCAATAAC
***ARTD15Y254ARev***	GTTATTGGTGACCACGAAGGCCTTGGGAGGGATGTCTC
***ARTD15qRT For***	GCCGTGTGTGAGGTCATTGA
***ARTD15qRT Rev***	TTGGAATCCTTCTTCTTGGTTTG
***GAPDHqRT For***	TGGGGCTCATTTGCAGGGG
***GAPDHqRT Rev***	TGGATGACCTTGGCCAGGG

Restriction sites are underlined.

### ARTD15 is a Mono-ADP-ribosyltransferase

The ARTD family members that lack conserved residues crucial for ADP-ribose polymer elongation have been proposed either to be inactive enzymes (ARTD9 and ARTD13) or to act as mono-ADP-ribosyltransferases. We thus analyzed whether ARTD15 was indeed an active enzyme.

To this end, we purified GST-ARTD15 from Escherichia coli. The recombinant GST-ARTD15 was tested in an in vitro ADP-ribosylation assay using [^32^P]-NAD. A main 50-kDa band, corresponding precisely to the molecular weight of purified GST-ARTD15 was detected (Fig. 4A). Thus, ARTD15 is able to catalyse its auto-ADP-ribosylation, a feature typical of all ARTC and ARTD enzymes. The GST-ARTD15 Km value for NAD was evaluated and found to be 290+/−47 µM, with a calculated Vmax of 0,7 pmole/h/µg of purified protein (Fig. 4B). To verify the specificity of this auto-modification, we generated a double GST-ARTD15 H152A/Y254A mutant (ARTD15-dm; H152 is involved in NAD binding and Y254 is the predicted catalytic amino acid). Figure 4C shows that the ARTD15-dm completely lacks the catalytic activity, as compared to the wild type protein. In conclusion, ARTD15 is an active enzyme that can catalyse its auto-ADP-ribosylation.

Next, we evaluated the activity of ARTD15 in mammalian cells by analysing both its ability to hydrolyse [^32^P]-NAD and to transfer the ADP-ribose moiety to agmatine, a well-known model substrate for arginine-specific mono-ADP-ribosylation (ARTC1). To this end, HeLa cells were transiently transfected with ARTD15; total cell membranes were then prepared and incubated with [^32^P]-NAD in the absence or presence of 1 mM agmatine. The supernatants were analysed by TLC to monitor the hydrolysis of the [^32^P]-NAD. Figure 4D shows that ADP-ribose is generated when [^32^P]-NAD was incubated with a NAD-glycohydrolase (NADase) (control; Fig. 4D left panel, NADase lane) but not when incubated with ARTD15 (Fig. 4D left panel, ARTD15 lane). As expected, agmatine was ADP-ribosylated by ARTC1 (Fig. 4D right panel), but not by ARTD15 (Fig. 4D, right panel). This implies that ARTD15 is an active enzyme, which however does not catalyse ADP-ribosylation on arginine; furthermore ARTD15 does not feature NADase activity per se and thus cannot affect NAD catabolism. In line with these conclusions, ARTD15 [^32^P]-ADP-ribosylation was not affected by the arginine-specific mono-ADP-ribosyltransferase inhibitor MIBG [47], as instead was the ADP-ribosylation of the Gß subunit, a well characterized target of arginine-specific mono-ADP-ribosyltransferases [2], [48], [49] (Fig. 4E).

To gain further insight as to which ARTD15 amino acid is modified by ADP-ribosylation, we investigated the chemical stability of the ADP-ribosyl linkage. NH_2_OH treatment for 12 h (which hydrolyzes the ADP-ribosylated arginine [14], [50], [51], [52]) did not remove the ADP-ribose of [^32^P]-ADP-ribosylated ARTD15 (Fig. 4F, lane 3); this is in agreement with the fact that ARTD15 is unable to modify agmatine (Fig. 4D, right panel) and that its activity is not inhibited by MIBG (Fig. 4E). The same experiment also ruled out the possibility that the ADP-ribose is linked to glutamate since a 20-min NH_2_OH treatment (normally sufficient to hydrolyze glutamate-linked ADP-ribose) did not remove ADP-ribose from ARTD15. Finally, treatment of [^32^P]-ADP-ribosylated ARTD15 with HgCl_2_ (which would act on ADP-ribosylated cysteine) was also ineffective. We did observe a 50% loss of label upon HCl treatment (Fig. 4F, lane 4), which is known to be effective on ADP-ribosylated serine/threonine residues [53].

Altogether, these data demonstrate that ARTD15 is an active member of the PARP family, with auto-catalytic activity; however, at variance with classical mono-ADP-ribosyltransferases it does not appear to target arginine or cysteine amino acid residues. Moreover, as opposed to PARP enzymes, ARTD15 is unable to transfer ADP-ribose onto glutamate. The specific aminoacid targeted by ARTD15 remains to be established.

### ARTD15 Interacts with Kapß1

Having provided evidence that ARTD15 is an active ADP-ribosyltransferase, we searched for possible interactors. To this end HEK293 cells were transiently transfected with a plasmid encoding for N-terminal tagged FLAG-ARTD15 or empty vector. Total lysates from these cells were immuno-precipitated with an anti-FLAG antibody (Fig. 5A). Two bands in the ARTD15 sample, with a molecular mass of 36 and 96 kDa, as revealed by silver staining (Fig. 5A) were identified by MALDI-TOF-MS analysis as ARTD15 itself and Kapß1 (Table 1). The same result was also obtained in HeLa cells (not shown). We validated the interaction between ARTD15 and Kapß1 by co-immunoprecipitation. Total lysates from HeLa cells that had been transiently transfected with FLAG-ARTD15 were incubated with anti-Kapß1 antibody (Fig. 5B). Under these conditions, ARTD15 was co-immunoprecipitated with endogenous Kapß1, demonstrating that Kapß1 is an ARTD15 interactor in intact cells. We could not detect co-immunoprecipitation of endogenous ARTD15, possibly due to the low levels of endogenous ARTD15.

To verify whether the interaction between ARTD15 and Kapß1 was direct, we applied a pull down assay using purified proteins. Increasing concentrations of Kapß1 were incubated with either GST-ARTD15 or GST alone as a control (Fig. 5C). Under these conditions, Kapß1 specifically interacted with GST-ARTD15 in vitro, providing evidence of a novel physical interaction between ARTD15 and Kapß1.

Finally, we evaluated this interaction in intact cells by immuno-fluorescence. HeLa cells were probed with antibodies against ARTD15 and Kapß1. Fig. 5D shows that indeed endogenous ARTD15 and Kapß1 co-localised, suggesting that Kapß1 most likely interacts with the cytosolic portion of membrane bound ARTD15.

### Kapß1 is a Target of ARTD15-mediated Mono-ADP-ribosylation

The finding that ARTD15 and Kapß1 are interacting partners, prompted us to investigate whether Kapß1 can be ADP-ribosylated by ARTD15. The enzymatic activity of ARTD15 towards Kapß1 was evaluated in an in vitro ADP-ribosylation assay using recombinant proteins. His-Kapß1 was incubated with or without GST-ARTD15 in the presence of 10 µM [^32^P]-NAD (Fig. 6A). Under these experimental conditions recombinant Kapß1 was specifically labelled only when incubated with ARTD15 (Fig. 6A). Thus, ARTD15 can ADP-ribosylate Kapß1. Moreover, His-Kapß1 ADP-ribosylated with biotinylated NAD was pulled-down with GST-ARTD15. We found that a similar percentage (ca 50%) of both unmodified His-Kapß1 and ADP-ribosylated His-Kapß1 interacted with ARTD15. To confirm that Kapß1 modification is a mono-ADP-ribosylation, and not a poly-ADP-ribosylation, we analyzed the length of the ADP-ribose chain bound to both ARTD15 and Kapß1 following a described procedure [54]. To this end, purified ARTD15 and Kapß1 were ADP-ribosylated as described above. The nucleotide fraction bound to the protein was separated by TBE-PAGE, with [^32^P]-NAD and [^32^P]-ADP-ribose used as reference standards (Fig. 6B). We found that the nucleotide fractions released from Kapß1 (lane 2–3) and from ARTD15 (lane 4) co-migrate with the single unit of ADP-ribose obtained from mono-ADP-ribosylated agmatine (lane 6), thus confirming that the ARTD15-catalysed reactions (both auto- and hetero-modifications) are very likely mono-and not poly-ADP-ribosylation events. ARTD15-mediated Kapß1 mono-ADP-ribosylation was further investigated making use of ADP-ribosylation inhibitors. Since we already knew MIBG to be ineffective, we tested the effects of PJ34, a well-characterized inhibitor of ARTD enzymes [55], and found that ARTD15 auto-modification and Kapß1 mono-ADP-ribosylation were both inhibited (Fig. 6C). Finally, the ability of ARTD15 to catalyze Kapß1 mono-ADP-ribosylation was also evaluated using isolated membranes, obtained from HeLa cells that had been previously transfected with ARTD15. Figure 6D shows that in the presence of ARTD15 a 96 kDa protein was specifically labelled. This same protein was recognized by an anti-Kapß1 antibody. We knocked-down endogenous ARTD15 in HeLa cells by siRNA and achieved a 70% decrease in ARTD15 levels; as a consequence, Kapß1 labelling decreased 45+/−12% (Fig. 6E). Altogether, these data confirm that ARTD15 and Kapß1 interact and that Kapß1 is specifically mono-ADP-ribosylated by ARTD15.

## Discussion

Here we report on the characterization of human ARTD15, unveiling a novel intracellular mono-ADP-ribosyltransferase. Endogenous mono-ADP-ribosylation is believed to have important roles in cellular signalling and regulation, however the enzymes responsible for this intracellular mono-ADP-ribosylation remain elusive and are only now beginning to be identified.

The human genome encodes 17 poly-ADP-ribose-polymerase (PARP)-like genes. Many of these PARP-like proteins (ARTDs according to the new nomenclature) however, are unlikely to carry out genuine ADP-ribose polymer formation; this is because although they do have a catalytic domain similar to that of ARTD1/PARP1, they lack the catalytic glutamate residue critical for polymerase activity [19], [20], [56]. A number of ARTDs (ARTD7, ARTD8 and ARTD12) have been proposed instead to act as cellular mono-ADP-ribosyltransferases [57], although only for ARTD10 has this been formally demonstrated [29]. This raises the possibility that other members of the PARP/ARTD family are intracellular mono-ADP-ribosyltransferases.

Here we show for the first time that ARTD15 is a bona fide mono-ADP-ribosyltransferase that catalyse auto- and hetero-modification by transfer of a single ADP-ribose moiety. ARTD15 has a Km for NAD around 290 µM for its own ADP-ribosylation, a value compatible with the intracellular NAD levels (ca. 500 µM). If we consider that these estimates were obtained in a reconstituted setting with the purified protein, it is conceivable that in physiological conditions kinetic parameters might be more favourable and affinity for NAD higher.

Of note, when we performed an in vitro ADP-ribosylation assay using total membranes obtained from cells over-expressing ARTD15, hetero-modification was the largely preferred reaction.

We also show that ARTD15 is a ubiquitous protein and appears to be the first ADP-ribosyltransferase known to be associated with the ER. While typical PARP enzymes are mainly nuclear, the most recently identified members are emerging as proteins having a mostly extra-nuclear localisation, as is the case of ARTD7, ARTD8 and ARTD12. These are cytosolic proteins that have been specifically identified as stress granule components, although their cellular functions and targets remain unknown [57]. An additional feature of ARTD15 is that it is the only ARTD family member with a carboxy-terminal transmembrane domain and an amino-terminal cytosolic catalytic domain. This orientation is shared with other known tail-anchored (TA) proteins and confirms a predictive bioinformatics study [58]. Several important protein families are TA-proteins, including the SNAREs, which mediate intracellular transport vesicle tethering, and the apoptotic regulator Bcl-2. The role of the tail anchor varies from protein to protein. In the case of SNAREs, for example, it is crucial for membrane fusion; in the Bcl-2 family, the tail anchor targets proteins to the mitochondrial outer membrane to control the release of apoptotic factors from the inter-membrane space [59]. The role of the ARTD15 tail anchor remains to be elucidated, but we can hypothesise that it might play a role in importin function, since we have identified Kapß1 as an ARTD15 interactor.

Kapß1 is a soluble receptor protein with a pivotal role in nuclear transport [60]. In our experiments, immunofluorescence analysis revealed that this protein co-localises with ARTD15 at the ER and the nuclear envelope. Kapß1 was recently found to associate with an ER-associated degradation (ERAD) transmembrane component (VIMP), thus supporting the hypothesis that Kapß1 plays a functional role at the ER [61]. We found that ER-resident ARTD15 catalyzes mono-ADP-ribosylation of Kapß1. This mono-ADP-ribosylation has some distinguishing features: i) it does not appear to occur on arginine, cysteine or glutamate residue. Although at this time the targeted aminoacid remains unknown, based on our findings we can speculate that ARTD15-mediatedADP-ribosylation might be occurring on a serine/threonine residue; this is at variance with the reactions catalysed by ARTC mono-ADP-ribosyltransferases and Sirt4, which specifically modify arginine and cysteine residues, respectively; ii) it is inhibited by PJ34, a well-characterized inhibitor of ARTD enzymes [55]. Since, however, PJ34 was described to act on the ß-NAD+ binding pocket of PARP1/ARTD1, we can hypothesise that it can also affect other ARTDs/PARPs having a similar NAD+ binding pocket structure, independently from whether the reaction catalyzed is a mono- or poly-ADP-ribosylation. iii) Finally, ARTD15-catalysed mono-ADP-ribosylation was not reverted by ARH1, which hydrolyzes the ADP-ribose-arginine bond, or by ARH3, which catalyzes the hydrolysis of poly-ADPribose and of *O*-acetyl-ADP-ribose (data not shown) [62], [63]. In conclusion, although the specific amino acid residue remain to be identified, the possibility exist that ARTD15 might be the first enzyme able to ADP-ribosylate a serine/threonine residue, a modification that had been described years ago [53]. Identification of the precise Kapß1 modification site will require further work and might be instrumental in defining the functional meaning of the ARTD15-Kapß1 interaction.

We suggest that ARTD15 might be a new player in the control of nuclear transport together with the nuclear pore complex, cargo proteins and karyopherins [64]. Two mechanisms have been described so far for the control of karyopherins: attenuation of protein expression and their sequestration. Indeed, it has been reported that poly-ADP-ribosylation of cargo proteins -but not of karyopherins- blocks their exit from the nucleus [65]. Mono-ADP-ribosylation of Kapß1 by ARTD15 might be a further level of control of nuclear transport. Future work will clarify whether ARTD15-mediated Kapß1 ADP-ribosylation can regulate Kapß1 interaction with its partners.

## Materials and Methods

### Cell Culture and Fractionation

Human embryonic kidney (HEK293; ATCC, CRL-1573), epidermoid carcinoma (A431; ATCC, CRL-1555), human leukemia (HL60; ATCC, CCL-240), prostate carcinoma (from a brain metastatic site; DU145; ATCC, HTB-81), ovary adenocarcinoma (SKOV-3; ATCC, HTB-77), colorectal carcinoma (HCT116; ATCC, ECL-247 and HT29; ATCC, HTB-38) and breast adenocarcinoma (MDA; ATCC, HTB-26) cells were grown in Dulbecco’s modified Eagle’s medium (DMEM; Invitrogen); cervix adenocarcinoma (HeLa; ATCC, CCL-2) cells were grown in Modified Eagle’s medium (MEM; Invitrogen); prostate carcinoma (from a bone metastatic site; PC3; ATCC, CRL-1435) and ovary adenocarcinoma (OVCAR-3; ATCC, HTB-161) cells were grown in RPMI medium; brain neuroblastoma (from a bone marrow metastatic site; SH-SY-5; ATCC, CRL-2266), melanoma (A375MM; obtained from Prof. Gustavo Egea, Universitat de Barcelona, Spain [66]) and kidney (HK2; ATCC, CRL-2190) cell lines were grown in DMEM:F12 (1∶1) medium (Invitrogen); all growth media were supplemented with 100 U/ml penicillin, 100 µg/ml streptomycin, 2 mM L-glutamine and 10% foetal bovine serum (FBS), all from Invitrogen. Total lysates, total membranes and cytosol fractions were prepared as previously described [14], [48]. Other reagents were purchased from Sigma-Aldrich, unless otherwise specified.

### Western Blotting and Antibodies

Cells were lysed and protein concentrations were determined with the Bio-Rad protein assay (Bio-Rad). Samples were separated by 10% SDS-PAGE and transferred to nitrocellulose filters (Perkin Elmer). Blots were incubated with primary antibodies diluted in T-TBS for 1 hour at room temperature or over-night at 4°C. After washes with T-TBS, blots were incubated in horseradish peroxidase (HRP)-conjugated secondary anti-mouse or anti-rabbit (Calbiochem) antibodies. Protein bands were visualised with the ECL-plus chemiluminescence reagent (GE Healthcare) according to manufacturer’s instructions. Densitometric evaluation was carried out with the public domain ImageJ software. The following primary antibodies were used: rabbit anti-ARTD15, mouse anti-GFP and rabbit anti-calnexin (C-ter) (AbCam), mouse anti-calnexin (N-ter; BD Bioscience), mouse anti-His, rabbit anti-actin, mouse anti-FLAG (Sigma-Aldrich), rabbit anti-biotin (Bethyl) and rabbit anti-GRK2 (Santa Cruz). We also used our own polyclonal antibodies against human ARTD15, rat GRASP55 and human GST, these were raised in rabbits using GST-ARTD15, His-GRASP55 and GST as immunogens, respectively. All were affinity-purified on their corresponding immunogens.

### Plasmids and Transfection

The pCMV-XL5 ARTD15 eukaryotic expression vector was purchased from OriGene. The PCR product corresponding to full length ARTD15 and ΔTM-ARTD15 (aa 1–277) was amplified from the pCMV-XL5-ARTD15 vector using forward *ARTD15HindFor* and reverse *ARTD15BamRev* or Δ*TM-ARTD15BamRev* primers (see Table 2). PCR products were gel-purified and subcloned into the *HindIII* (5′) and *BamHI* (3′) sites of the p3xFLAG-CMV-10, which encodes the FLAG-tag at the N-terminus. To obtain GFP-tagged constructs, the full-length ARTD15 PCR product obtained using *ARTD15EcoFor* and *ARTD15SalRev* primers (see Table 2) was subcloned into the *EcoRI* (5′) and *SalI* (3′) sites of pEGFP-C3 and pEGFP-N2 vectors (Clontech). Restriction enzymes were all from New England Biolabs. HeLa and HEK293 cells were transfected with the different cDNAs using TransIT-LT1 transfection reagent (MirusBio) according to manufacturer’s instructions. To silence ARTD15, Hela cells were transfected with a pool of two siRNAs (50 nM), using Lipofectamine 2000 (Invitrogen). The following siRNAs (Qiagen) were used: 1) AACAGTCATGTTTCTCATAAA; 2) TACAGCTGAAATGGAACCAAA. 70% of endogenous ARTD15 was knocked down after 72 h, as evaluated by WB. As control, the following Scrambled oligos (Sigma) were used: 1) GCTTCCTACCACAATTTCT; 2) GGTAATCAACTAATCTTAA.

### Site-directed Mutagenesis

Point mutations were generated using Quik Change Site-directed mutagenesis kits (Stratagene), following the manufacturer’s instructions. Double mutagenesis was performed on the pCMV-XL5-ARTD15 plasmid, using the oligonucleotides ARTD15(H152A)For and ARTD15(H152A)Rev, and then on the pCMV-XL5-ARTD15H152A plasmid, using the oligonucleotides ARTD15(Y254A)For and ARTD15(Y254A)Rev. The oligonucleotide sequences are reported in Table 2. The construct sequences were confirmed by automated DNA sequencing.

### Protease Protection Assay

HeLa cells were plated in 12-well dishes and then transfected with pEGFP-N3-ARTD15 or pEGFP-C2-ARTD15. Twenty-four hours later, cells were washed with PBS and analyzed as previously described [58], with minor modifications. Monolayers were either left untreated or permeabilised with digitonin (100 µg/ml) in 25 mM Tris/250 mM sucrose pH 7.4 for 1,5 minutes. The digitonin-containing solution was then removed and replaced with trypsin (120 µg/ml) in 25 mM Tris/250 mM sucrose pH 7.4 for 3 minutes. Alternatively, cells were permeabilised with 1% Triton X-100 in 25 mM Tris/250 mM sucrose pH 7.4 with trypsin (120 µg/ml) for 3 minutes. Proteolysis was blocked by adding complete protease inhibitors (Roche) at 10 fold the recommended concentration with the addition of 1 mM phenylmethylsulfonyl fluoride (PMSF). Total cell lysates were prepared in RIPA buffer, separated by SDS-PAGE and immunoblotted.

### RNA Extraction

RNA was isolated from the various cell lines using TRIzol reagent (Invitrogen) according to the manufacturer’s instructions. RNA samples were dissolved in water and quantified with a spectrophotometer at 260 nm. Samples were treated with DNase-I (Ambion). Total RNA (2 µg) was retro-transcribed using the Enhanced Avian RT First Strand Synthesis Kit according to manufacturer’s instructions (Sigma-Aldrich).

### Quantitative Real-time PCR (qRT-PCR)

qRT-PCR was performed as described [67]. Primer sequences for human ARTD15 and GAPDH genes (Table 2) were designed using Primer Express 3.0 software (Applied Biosystems). Quantitative normalization of the cDNA in each sample was carried out using glyceraldeyde-3-phosphate dehydrogenase (GAPDH, accession number: X52123.1) amplification as internal control. Relative quantification was done using the comparative ΔC_T_ method.

### Recombinant Protein Purification

To obtain the N-terminally GST-tagged construct, purified full-length ARTD15 and ARTD15-H152A/Y254A (ARTD15-dm) PCR products were subcloned into the pGEX-4T1 vector (GE Healthcare) using the *EcoRI* (5′) and *SalI* (3′) sites. The pQE60-Kapß1 vector encoding full-length His-Kapß1 was kindly provided by Prof. Gino Cingolani (Department of Biochemistry and Molecular Biology, Jefferson Medical College, Philadelphia, USA). For GST-ARTD15 (wt and dm) production, transformed DH5α cells were grown in Luria-Bertani medium at 37°C, induced with 0.1 mM isopropyl-d-thiogalactoside (IPTG), and grown for a further 16–20 h at 20°C. Purification of recombinant GST-tagged ARTD15 (wt and dm) from the soluble lysate protein was accomplished with Glutathione Sepharose 4B resin (GE Healthcare) following the manufacturer’s instructions. For His-Kapß1 purification transformed BL21 (DE3) cells (Novagen) were grown in Luria-Bertani medium at 37°C, induced with 0.5 mM IPTG, and grown for a further 16–20 hours at 37°C. Purification of recombinant His-tagged Kapß1 protein from the soluble lysate protein was accomplished with the Ni-NTA (Qiagen) affinity resin following the manufacturer’s instructions.

### Immunoprecipitation, GST-pull-down and MALDI-ToF Analysis

HeLa or HEK293 cells were transfected with FLAG-ARTD15. After 24 hours, cells were lysed in IP buffer (25 mM Tris-Hcl, pH 7.5, 150 mM NaCl, 1% Triton X-100, 5 mM EDTA, 5 mM MgCl2, 1 mM DTT, 1 mM NaVO4, 40 mM ß-glycerophosphate) with protease inhibitors. The lysates were collected and centrifuged at 10.000 g for 10 min at 4°C. The supernatants were then diluted to 0.2% Triton X-100 final concentration. For ARTD15 immunoprecipitation, total lysates from HeLa or HEK293 cells (6 mg) were incubated over-night at 4°C with constant rotation with anti-FLAG antibody (0.5 µg/mg lysate) or control IgG. The samples were then incubated with a 50% protein-A sepharose resin slurry for 2 h at 4°C with constant rotation. Resins were centrifuged, washed five times and eluted with Laemmli buffer. Eluates were separated by SDS-PAGE and analysed by Silver staining. Differential protein bands were cut, trypsinised and analysed by MALDI-TOF mass spectrometry. For Kapß1 immunoprecipitation, total lysates (2 mg) from HeLa cells over-expressing FLAG-ARTD15 were incubated with an anti-Kapß1 antibody (1 µg/mg lysate) or with the same amount of control IgG over-night at 4°C with constant rotation. The day after, the samples were incubated with 50 µl of a 50% protein-A sepharose resin slurry for 2 h at 4°C with constant rotation. The samples were then centrifuged and resins were washed 5 times in IP buffer with 0.2% Triton X-100, and one time using IP buffer without detergent. Resins were then eluted with 100 µl of Laemmli buffer, boiled for 5 minutes at 100°C and separated by 12% SDS-PAGE. Proteins were transferred to nitrocellulose membrane for immunoblotting.

The GST-pull-down experiments were performed as previously described (Cai et al., 2007). Briefly, His-Kapß1 was incubated over-night at 4°C with GST or GST-ARTD15 protein in binding buffer (50 mM Tris-Hcl, pH 7.4, 100 mM NaCl, 0.1% Triton X-100, 0.1% NP-40 and 1 mg/ml BSA). The samples were then incubated with a 50% Glutathione Sepharose 4B resin slurry for 2 h at 4°C with constant rotation and centrifuged at 500 g for 5 minutes. The resin was washed 5 times with washing buffer (50 mM Tris-Hcl, pH 7.4, 100 mM NaCl) and eluted with Laemmli buffer. The eluates were analysed by SDS-PAGE and Western Blot.

### ADP-ribosylation Assay, Thin Layer Chromatography (TLC) and ADP-ribosylation Product Analysis

ARTD15 activity was measured by using a previously described [^32^P]-ADP-ribosylation assay [14], [48]. ADP-ribosylation with biotin-NAD (25 µM; 6-biotin-17-NAD Trevigen) was performed as previously described [68]. The kinetic parameters were determined by incubating 300 ng of purified GST-ARTD15 with a fixed amount of [^32^P]-NAD^+^ (2 µCi) and increasing concentration of cold NAD (1–1000 µM) for 16 h at 37°C. The [^32^P]-ADP-ribosylated ARTD15 was quantified with an InstantImager (Packard Instrument Co). Thin-layer chromatography was performed as described [67]. Nucleotide were released from ADP-ribosylated proteins as described previously [54], [69] with a minor modification: the TCA-precipitated samples were incubated in 100 µl of 10 mM Tris-NaOH, pH 12, 1 mM EDTA and 0,2 mg/ml proteinase K at 60°C for 3 hours. The length of ADP-ribosylation products was analysed by high-resolution gel electrophoresis, as described previously [54].

### ADP-ribose Bond Stability

ADP-ribose-ARTD15 bond stability was examined as described previously [70], with minor modifications. GST-ARTD15 was [^32^P]-ADP-ribosylated and electroblotted; radioactivity on the filter was quantified with an InstantImager. The filter was then divided into separate strips, each of which was incubated with 50 mM Hepes, 1% SDS containing either 1 mM HgCl_2_ at 37°C for 1 hour, or 0.5 M NH_2_OH at 37°C for 20 minutes, or 1 M NH_2_OH at 37°C for 12 hours, or 0.2 M HCl at 37°C for 2 hours or 1 M NaCl at 37°C for 12 hours. After treatments the filters were washed twice with water for 5 minutes and analyzed with an InstantImager.

### Indirect Immunofluorescence and Antibodies

HeLa cells were grown on coverslips in 24-well plates and transfected with TransIT-LT1 (MirusBio, USA). Immunofluorescence was performed as described [67]; using an anti-FLAG antibody (ANTI-FLAG® M2 antibody, F3165 Sigma; 1∶500 in blocking solution) and an anti-ARTD15 antibody (AbCam; 1∶50 in blocking solution) to stain ARTD15. Anti-PDI (SPA-891 Stressgen; 1∶200 in blocking solution); anti-calnexin (BD Bioscience; 1∶200 in blocking solution); and anti-Kapß1 (AbCam; 1∶500 in blocking solution) antibodies were also used as primary antibodies. Alexa 488- and Alexa 546-conjugated goat anti-rabbit and anti-mouse IgG (Molecular Probes; 1∶400 in blocking buffer) were used as secondary antibodies. Samples were analysed using a confocal microscope (Zeiss LSM 510).

### Electron Microscopy

HeLa cells transfected with FLAG-ARTD15 were used for immuno-EM analysis using the gold-enhance protocol [71] and cut as described previously [72]. Briefly, cells were fixed with 4% formaldehyde and 0.05% glutaraldehyde for 10 min at 37°C, and postfixed for 30 min with 4% formaldehyde alone, at room temperature. After washing with PBS and a 20-min treatment with a blocking solution containing 1% bovine serum albumin, 50 mM NH4Cl and 0.2% saponin, samples were incubated with a primary rabbit anti-FLAG antibody for 1 h. Samples were then incubated for 2 h with nanogold-conjugated anti-rabbit IgG (Nanoprobes) diluted in blocking solution (1∶100) and extensively washed. Gold particles were enhanced with Gold Enhancer (Nanoprobes), according to the manufacturer’s instructions. Samples were then fixed with 1% glutaraldehyde in 0.15 M HEPES (pH 7.3) at 37°C for 5 min. After fixation, the cells were scraped and collected in 1.5 ml tubes. Collected cells were centrifuged and treated with 1% OsO4 plus 1.5% potassium ferrocyanide in 0.1 M cacodylate buffer (pH 7.3) for 1.5 h at room temperature in the dark. Samples were then dehydrated by consecutive washes with ethanol at increasing concentrations (50%–70%–90%–100%) and finally embedded in Epon 812. Finally, samples were examined on a Tecnai 12 electron microscope at 120 kW (FEI/Philips Electron Optics).
